# Incidence of osteoporosis and ambient air pollution in South Korea: a population-based retrospective cohort study

**DOI:** 10.1186/s12889-021-11866-7

**Published:** 2021-10-06

**Authors:** Jinyoung Shin, Hyuk Jung Kweon, Kyoung Ja Kwon, Seol-Heui Han

**Affiliations:** 1 Department of Family Medicine, Konkuk University Medical Center, Konkuk University School of Medicine, Seoul, South Korea; 2 Department of Family Medicine, Konkuk University Medical Center, Chungju Hospital, Konkuk University School of Medicine, Chungju, South Korea; 3 Department of Neuroscience, Konkuk University School of Medicine, Seoul, South Korea; 4 Department of Neurology, Konkuk University Medical Center, Konkuk University School of Medicine, 120-1 Neungdong-ro, Gwangjin-gu, Seoul, 05030 South Korea

**Keywords:** Air pollution, Particulate matter, Osteoporosis, Epidemiology

## Abstract

**Background:**

This study investigated the associations between exposure to ambient air pollutants and the incidence of osteoporosis using the Korean National Insurance Service–National Sample Cohort.

**Methods:**

This nationwide, population-based, retrospective cohort study included 237,149 adults aged ≥40 years that did not have a diagnosis of osteoporosis at baseline between January 1, 2003, and December 31, 2015. Osteoporosis was defined as claim codes and prescriptions of bisphosphonates or selective estrogen receptor modulators at least twice annually. After matching values for PM_10_, NO_2_, CO, and SO_2_ during the 2002–2015 time period and PM_2.5_ in 2015 with residential areas, the incidence of osteoporosis was analyzed using a Cox proportional hazards regression model according to the quartile of average yearly concentrations of pollutants.

**Results:**

Overall 22.2% of the study subjects, 52,601 (male: 5.6%, female: 37.6%) adults in total, were newly diagnosed with osteoporosis and treated. Exposure to PM_10_ was positively associated with incidence of osteoporosis (Q4: 1798 per 100,000 person-years vs. Q1: 1655 per 100,000 person-years). The adjusted hazard ratio (HR) with 95% confidence interval (CI) of Q4 in PM_10_ was 1.034 (1.009–1.062). The effect of PM_10_ on osteoporosis incidence was distinct in females (adjusted sub-HR: 1.065, 95% CI: 1.003–1.129), subjects aged < 65 years (adjusted sub-HR: 1.040, 95% CI: 1.010–1.072), and for residents in areas with low urbanization (adjusted sub-HR: 1.052, 95% CI: 1.019–1.087). However, there was no increase in osteoporosis based on exposure to NO_2_, CO, SO_2_, or PM_2.5_.

**Conclusions:**

Long-term exposure to PM_10_ was associated with newly diagnosed osteoporosis in Korean adults aged ≥40 years. This finding can aid in policy-making that is directed to control air pollution as a risk factor for bone health.

**Supplementary Information:**

The online version contains supplementary material available at 10.1186/s12889-021-11866-7.

## Background

Osteoporosis is a systemic skeletal disorder characterized by decreased bone mass and deteriorated bone architecture [1]. Osteoporosis is expected to progress continuously with age. A Korean longitudinal 12-year follow-up study reported that the prevalence of osteoporosis increased from 48.4 to 66.1% [2]. In 2008, the proportion of patients with osteoporosis who were aged over 50 years was 20.7% (6.1% among men and 33.3% among women) in the Korean general population. In 2008–2011, the associated health costs increased from USD 3.98 billion to USD 5.13 billion, with an annual increase of 9.2% [3, 4]. Osteoporosis also can lead to fragility fractures, resulting in poor health-related quality of life, high risk of mortality, and increased medical costs [5, 6].

Some of the well-known risk factors for osteoporosis are malnutrition, lifestyle, medications such as corticosteroids, and inherent characteristics such as old age and female sex [7]. Exposure to ambient air pollution, which includes particulate matter (PM) ≤10 μm (PM_10_) and ≤ 2.5 μm (PM_2.5_) in size, nitrogen dioxide (NO_2_), carbon monoxide (CO), and sulfur dioxide (SO_2_), has been recognized as a serious medical issue. Several studies have shown a relationship between air pollution and osteoporosis [8–12]. Ten-year exposure to CO and NO_2_ in 36,608 Taiwanese individuals and to PM and NO_2_ in 8033 Chinese rural populations was reported to increase the risk of osteoporosis [8, 9]. However, the associations between air pollutants and the incidence of osteoporosis were heterogeneous according to age, sex, exposure duration, or bone health indicators [10, 11]. In the Oslo Health Study from 2000 to 2001, osteoporosis was not associated with air pollution in women and men 59 to 60 years of age [10]. Air pollution was associated with vitamin D deficiency but not with bone-turnover markers in serum of Tehran adolescents [11]. Long-term exposure to PM_2.5_ and PM_10_ among 590 Norwegian older men increased the risk of decreased bone mineral density (BMD) [12]. However, the associations in different ages or women are unknown because the study only evaluated men born between 1924 and 1925. Therefore, there is a need for nationwide population-based studies that include the general population.

This study aimed to determine the association between long-term exposure to air pollutants and newly diagnosed osteoporosis using a national database.

## Methods

### Data sources

This was a nationwide, population-based, retrospective, cohort study that used data from the Korean Health Insurance Service (NHIS)–National Sample Cohort (NHIS–NSC). The mandatory social National Health Insurance Service includes almost the entire Korean population (97.2%, approximately 50 million individuals). For this study, we used NHIS–NSC data with a systematic stratified random sample of 1 million people (representing approximately 2% of the total population in 2002, *n* = 1,025,340), with proportional allocation based on administrative district according to participant age (18 groups), sex (male, female), income level (41 groups), and total annual medical expenses [13]. The cohort consisted of residence distribution across 16 regions in Korea from 2002 to 2013. During the follow-up years in the sample cohort, the change in initial residence was 0–0.3% [13]. The NHIS claims data included information on diagnosis, procedures, and prescriptions, as identified by the International Classification of Diseases, Tenth Revision (ICD-10) codes, and by the Korean Drug and Anatomical Therapeutic Chemical Codes. This study was approved by the Institutional Review Boards (IRBs) of the Clinical Research Ethics Committee of Konkuk University Medical Center, Seoul, Korea (KUH 2019–05-017).

### Study participants

**Fig. 1:**
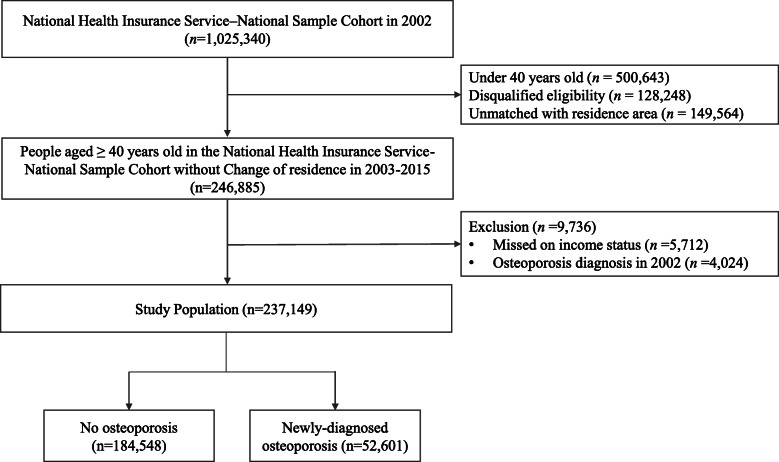
Flow chart of study population

We included 246,885 participants after excluding those younger than 40 years in the NHIS–NSC data in 2002 (*n* = 500,643), those with a residence change during 2002–2015 (*n* = 149,564), and those determined to be ineligible due to factors such as death or emigration (*n* = 128,248), because the NHIS–NSC replaces disqualified people with infants. A total of 237,149 individuals was included in the final analysis after excluding participants with missing data on income level (*n* = 5712) and those who had an osteoporosis diagnosis in 2002 (*n* = 4024) (Fig. 1).

Index date was defined as the date of the first diagnosis of osteoporosis. Osteoporosis was defined as a confirmed diagnosis by a physician (ICD-10; M80–M82) and subsequent treatment with either bisphosphonate such as alendronate, risedronate, ibandronate, or zoledronate or selective estrogen receptor modulator (SERM) such as raloxifene or bazedoxifene, at least twice annually between January 1, 2003, and December 31, 2015.

### Air pollutant variables

We determined the average concentrations of PM_10_, NO_2_, CO, and SO_2_ measured every hour from 2002 to 2015, at the Korean Air Pollutants Emission Service. Because PM_2.5_ has been measured in Korea since 2015, we used the average concentration in 2015. The air pollutants were measured at 268 nationwide surveillance stations, which cover most living areas of the South Korean population, except mountains or green areas. Residential five-digit codes that were designated as “Si,” “Do,” “Gun,” and “Gu” were used to match the location of the air pollution surveillance stations. We calculated the quartile of average yearly concentrations of all pollutants.

PM_10_ and PM_2.5_ were measured using a β-ray attenuation system (PM − 711D, DONGIL GREENSYS, Seoul, Korea). NO_2_ was measured using a chemiluminescence instrument (CM2041, APM ENGINEERING CO., LTD, Gyeonggi–do, Korea). CO was measured using a non-dispersive infrared sensor (ZKJ, DONGIL GREENSYS, Seoul, Korea). SO_2_ was measured using an ultraviolet (UV) fluorescence system (CM2050, APM ENGINEERING CO., LTD, Gyeonggi–do, Korea). Measurements of all air pollutants were performed according to the standard operating procedure of the Korean Air Pollutants Emission Service of the National Institute of Environmental Research (Incheon, South Korea). The levels of air pollutants and data for meteorological parameters (2003–2015), including annual average temperature, total rainfall, and wind speed, are presented in Table 1.

**Table 1:** Air pollutants and meteorological data

Variables	Mean	SD	IQR	Percentiles				
				Minimum	25th	50th	75th	Maximum
Air pollution								
PM_2.5_, μg/m^3^	23.1	4.4	7.0	16.0	20.3	22.0	27.3	30.0
PM_10_, μg/m^3^	53.0	4.9	9.1	44.8	48.5	52.4	57.6	61.0
CO, 10 ppm	5.9	0.7	1.1	5.2	5.3	5.6	6.4	7.0
SO_2_, ppb	5.7	0.5	0.7	4.7	5.3	5.7	6.0	6.4
NO_2_, ppb	25.3	1.9	2.1	23.0	24.0	25.0	26.1	29.1
Annual average weather conditions								
Temperature, °C	12.8	0.6	1.1	12.0	12.2	12.9	13.3	13.6
Rainfall, mm	1486	395	444	792	1320	1451	1764	2044
Wind speed, m/s	2.48	0.24	0.3	2.0	2.4	2.45	2.7	2.8

*SD* Standard deviation, *IQR* Interquartile range. Levels of particulate matter < 10 μm (PM_10_), sulfur dioxide (SO_2_), nitrogen dioxide (NO_2_), and carbon monoxide (CO) were measured between 2002 and 2015. Particulate matter < 2.5 μm (PM_2.5_) was measured in 2015. Temperature, rainfall, and wind speed were measured between 2003 and 2015. Korea Meteorological Administration, Seoul, Korea (https://data.kma.go.kr/climate/extremum/selectExtremumList.do?pgmNo=103)

### Other variables

Age was calculated from the participant’s birth year until 2002. Insurance units were divided into 11 categories (10: national health insurance, one: medical aid) based on income status and was divided into two groups (low: 1–5 and medical aid, high: 6–10) and considered as income divisions. The changes in average health insurance level were negligible during the study period [13]. The residential area was classified into two groups for subgroup analysis: 1) Seoul and the six biggest cities were classified as “high urbanization” areas, and 2) other areas were classified as “less urbanization.” The following significant comorbidities related to osteoporosis were surveyed based on physician diagnosis before the first diagnosis of osteoporosis using ICD-10 codes: peptic ulcer disease (K25), diabetes mellitus (E10–E14), cerebrovascular disease (I63, I64), peripheral vascular disease (I73), chronic pulmonary disease (J44), congestive heart failure (I50), myocardial infarction (I21, I22), malignancy including solitary organ, leukemia, and lymphoma (C00–C97), liver disease (K74), hemiplegia (G81–G83), and chronic kidney disease (N18). Mental disorders were classified as sensitive information and masked (F*). Therefore, based on the sensitive and masked information, mental disorders could not be differentiated in detail.

### Statistical analyses

Continuous variables are presented as mean with standard deviation, and categorical variables are presented as number and percentage. We compared the characteristics of study subjects between the osteoporosis and non-osteoporosis groups using the t-test and chi-square test. We calculated the hazard ratio (HR) and 95% confidence interval (CI) for newly diagnosed osteoporosis cases using the Cox proportional hazard regression model after adjusting for age, sex, income, urbanization, comorbidities, and meteorological data. We also calculated p-interaction values to compare the subgroup-HR for age (divided by 65 years), sex (male and female), and urbanization (high: Seoul and the six biggest cities in Korea, and low: the other areas) to find an effect modification of these risk factors by adding an interaction term (level of risk factor [age, sex, and residence] X presence of osteoporosis). All analyses were performed using SAS software 9.4 (SAS Institute Inc., Cary, NC, USA), and *P* < 0.05 was considered to indicate statistical significance.

## Results

The characteristics of the 237,149 subjects enrolled in this study are shown in Table 2. During the observation period, 52,601 patients with osteoporosis (22.2%) were newly diagnosed and treated. The incidence rate was 5.6% in males and 37.6% in females. The osteoporosis patient group was older and had a higher proportion of females, less urbanization, and more comorbidities than the non-osteoporosis group. However, there was no significant difference in income level according to diagnosis of osteoporosis.

**Table 2:** Baseline characteristics (*n* = 237,149)

	No osteoporosis (*n* = 184,548)	Osteoporosis (*n* = 52,601)	*P*- value
Age, year	50.1 ± 8.7	56.7 ± 9.6	< 0.001
Sex			< 0.001
Male	107,764 (94.4)	6394 (5.6)	
Female	76,784 (62.4)	46,207 (37.6)	
Income levels			0.824
Lower	58,269 (77.8)	16,635 (22.2)	
Upper	126,279 (77.9)	35,966 (22.1)	
Urbanization			< 0.001
High	87,979 (79.5)	22,723 (20.5)	
low	96,569 (76.4)	29,878 (23.6)	
Peptic ulcer disease	22,698 (12.3)	10,236 (19.5)	< 0.001
Diabetes mellitus	20,930 (11.3)	8479 (16.1)	< 0.001
Cerebrovascular disease	3555 (1.93)	1988 (3.78)	< 0.001
Peripheral vascular disease	3146 (1.70)	1981 (3.77)	< 0.001
Chronic pulmonary disease	1727 (0.94)	926 (1.76)	< 0.001
Mental disorder	1711 (0.93)	1042 (1.98)	< 0.001
Congestive heart failure	790 (0.43)	544 (1.03)	< 0.001
Myocardial infarction	513 (0.28)	188 (0.36)	0.003
Malignancy	391 (0.21)	159 (0.30)	< 0.001
Liver disease	379 (0.21)	131 (0.25)	< 0.001
Hemiplegia	354 (0.19)	147 (0.28)	< 0.001
Chronic kidney disease	304 (0.16)	101 (0.19)	0.181

Data were presented as mean ± standard deviation or number (percentage). *P* values were obtained by t test or chi-square test

Table 3 shows the incidence rate per 100,000 person-years, HR, and 95% CI for newly diagnosed osteoporosis cases according to the levels of air pollutants. Higher concentrations of PM_10_ were associated with increased incidence of osteoporosis (*p* = 0.021), but the result was not dose dependent. The HRs (95% CI) of osteoporosis based on exposure to NO_2_ and SO_2_ increased, but a significant difference was not observed. No association was found between exposure to CO or PM_2.5_ and osteoporosis.

**Table 3:** The hazard ratios (HRs) of osteoporosis incidence according to the levels of air pollutants

Quartile for air pollutants		Case/participants	Annual incidence rate per 100,000	Total (*n* = 237,149)			
				HR	95% CI		P for trend
					Lower	Upper	
PM_10_	Q1	12,805/59,520	1655	1			0.021
	Q2	12,450/56,657	1690	1.034	1.009	1.062	
	Q3	12,699/58,297	1682	1.023	0.997	1.050	
	Q4	14,647/62,675	1798	1.035	1.009	1.063	
NO_2_	Q1	11,270/56,710	1529	1			0.602
	Q2	11,047/55,175	1540	1.005	0.981	1.029	
	Q3	13,423/58,788	1756	1.015	0.988	1.042	
	Q4	16,861/66,476	1951	1.021	0.992	1.051	
SO_2_	Q1	12,542/57,916	1666	1			0.062
	Q2	13,523/59,347	1753	0.998	0.975	1.022	
	Q3	12,249/58,741	1604	0.976	0.951	1.000	
	Q4	14,287/61,145	1797	1.001	0.974	1.026	
CO	Q1	12,324/56,262	1685	1			0.753
	Q2	13,856/61,355	1737	1.001	0.977	1.026	
	Q3	14,176/62,220	1753	1.007	0.981	1.034	
	Q4	12,245/57,312	1643	0.998	0.971	1.023	
PM_2.5_	Q1	748/52,226	1433	1			0.506
	Q2	718/51,355	1399	0.993	0.965	1.022	
	Q3	957/68,450	1398	0.983	0.956	1.010	
	Q4	984/65,118	1511	1.007	0.985	1.029	

HRs and 95% confidence interval (CI) were obtained by the Cox-proportional hazard model after adjusting for age, sex, income levels, urbanization, comorbidities, and meteorological data.

**Fig. 2:**
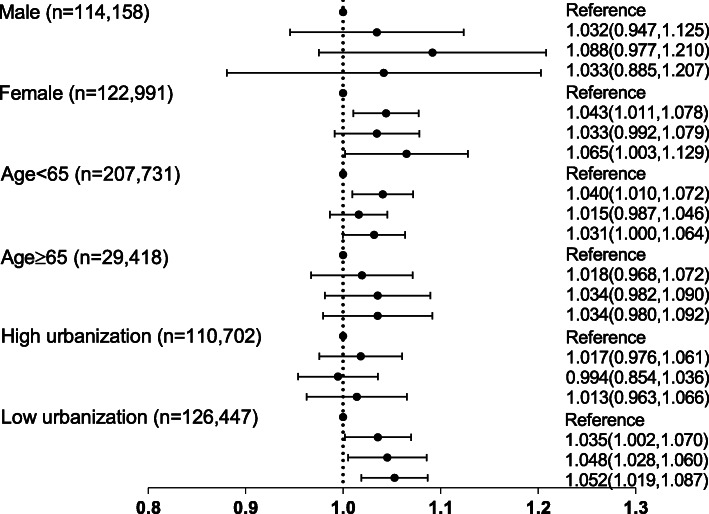
Adjusted sub-hazard ratios (HRs) and 95% confidence interval (CI) for osteoporosis incidence according to the quartile of PM_10_. HRs and 95% CI were obtained by the Cox-proportional hazard model after adjusting for age, sex, insurance level, urbanization, and comorbidities (selective stratification variable was not included in the model)

Figure 2 shows the association between PM_10_ and newly diagnosed osteoporosis according to sex, age (65 years), and urbanization. We found an association between osteoporosis and quartile of PM_10_ in female subjects (adjusted sub-HR [Q4]: 1.065, 95% CI: 1.003–1.129), those aged < 65 years (adjusted sub-HR [Q2]: 1.040, 95% CI: 1.010–1.072), and those of low urbanization (adjusted sub-H R [Q4]: 1.052, 95% CI: 1.019–1.087). P-interaction values were sex (*p* = 0.007), age (divided at 65 years, *p* = 0.015), and urbanization (< 0.001).

## Discussion

This nationwide, retrospective, population-based Korean cohort study used claims data and found that long-term exposure to PM_10_ was positively associated with incidence of osteoporosis. Notably, we confirmed that the effect of PM_10_ on newly diagnosed osteoporosis was more evident in females, subjects aged < 65 years, and those who resided in a non-metropolitan area (low urbanization). The association between air pollution and osteoporosis has not been studied widely because it has been considered an intrinsic factor [14]. However, this study confirmed that exposure to air pollutants might be an independent risk factor for osteoporosis in the general population.

Bones can serve as a reservoir for sequestration of air pollutants [14]. Therefore, pollutants directly can affect the bone and have an indirect effect through decreased vitamin D level, decreased osteoblasts, and increased osteoclasts [14]. Air pollution can absorb Ultraviolet B (UVB) photons that can restrict the amount of solar UVB radiation reaching the Earth’s surface. Air pollution also diminishes the cutaneous photosynthesis of vitamin D by elevating parathyroid hormone levels, increasing bone resorption, and decreasing total body BMD [15, 16]. A low air quality index in Isfahan, Iran, was associated with reduced UVB radiation and vitamin D deficiency in young children despite the region having high sunlight exposure [17]. We confirmed that PM_10_ was the only significant source of air pollution related to incidence of osteoporosis in Korean adults aged ≥40 years. One report indicated that PM concentrations directly decreased the serum level of parathyroid hormone and BMD in 692 middle-aged men in the USA [18]. Our study could not show a causal relationship between PM_10_ and osteoporosis because we were not able to access individuals’ serum vitamin D or parathyroid hormone levels in the claims data; however, based on the results from previous studies, we hypothesize underlying mechanisms [15–18].

One important finding that often is neglected is consideration of differences for patients with chronic renal failure, which impacts vitamin D metabolism, although the result was not statistically significant [16, 19]. The effects of vitamin D metabolism on renal function cannot be concluded but also cannot be ruled out. One study reported that renal function was not a risk factor for osteoporosis after adjusting for age and other confounding factors in 776 postmenopausal Chinese women, similar to the results of our study [20]. Therefore, we can consider the independent role of vitamin D, not through kidney dysfunction, in the association between exposure to air pollutants and incidence of osteoporosis. A cross-sectional US population-based study indicated that the parathyroid hormone levels did not differ according to amount of air pollution [11]. In addition, exposure to air pollutants can lead to increased systemic inflammation through specific pro-inflammatory cytokines such as tumor necrosis factor-α, interleukin (IL)-1β, IL-6, and IL-17, which affect osteoblast and osteoclast differentiation and function [21, 22]. An increase in oxidative damage with advancing age represents a general pathophysiological mechanism for age-related osteoporosis [23]. However, osteoporosis that results from exposure to air pollutants can increase further in those younger than 65 years due to frequent social engagements compared to older people aged 65 or older. Therefore, it is necessary to carefully interpret the mechanism for these findings based on further research on the association between air pollution and incidence of osteoporosis.

In this study, only PM_10_ among the surveyed pollutants showed an association with incidence of osteoporosis. There are two possible reasons for this association. First, an area-based approach, which assigned exposures at the level of participant community, county, postcode, or census tract, might not be suitable for evaluating the effects of NO_2_, SO_2_, and CO, which are affected by local sources and exhibit small-scale gradients in concentrations compared to that of PM, which has a relatively homogenous dispersion across space [24]. Second, the concentration of NO_2_ determined in our study might be too low to have a sufficient effect on incidence of osteoporosis. The NO_2_ level reported in the Taiwan study, which showed evidence of an association between NO_2_ level and osteoporosis incidence, was higher than the NO_2_ level determined in our study (highest quartile: > 9825.1 ppb in the Taiwan study vs. > 26.1 ppb in our study) [8].

PM_2.5_ has lower source contributions from soil dust and natural sources than does PM_10_. It also has higher source contributions from secondary aerosol sources due to atmospheric chemical reaction of gaseous pollutants such as SO_2_ and NO_2_, which are associated with greater health hazards [25]. However, PM_2.5_ showed no association with incidence of osteoporosis. This result is likely because the mean PM_2.5_ level was lower in this study than in the study in rural China that showed a correlation with incidence of osteoporosis (mean ± SD: 23.1 ± 4.4 μg/m^3^ vs. 72.1 ± 1.9 μg/m^3^) [9]. The reason for this finding might be a threshold effect at a low level of air pollutants or that the association between PM and osteoporosis might not be clear or the relationship might be non-linear particular cut-off values of air pollutants [26]. Furthermore, the results might not have reflected the regional deviation for that year because we only measured the average level of PM_2.5_ in 2015, and observed the incidence of osteoporosis in the same period. Therefore, we cannot confirm if PM_2.5_ is associated with osteoporosis, and future research should reassess the associations for more than 1 year.

An increased risk of osteoporosis with higher air pollution levels was observed in female subjects, while there was no association in male subjects. Although little is known about the effect on sex in the association between air pollutants and osteoporosis, the reason for the difference between males and females is attributable to decreased estrogen level due to air pollutants can cause bone loss by upregulation of receptor activator of nuclear factor kappa-B ligand (RANKL), osteoblast proliferation, or osteoprotegerin production [21]. In contrast with the results of this study, the Oslo Health Study showed no association between long-term exposure to PM_10_ and PM_2.5_ from 1992 to 2001 in women and negative associations in men aged 75 or 76 years [10]. Because our study was conducted and focused on the incidence of osteoporosis, the decreased estrogen effects caused by air pollutants might have a strong influence in menopausal women rather than women in their 70s, as reported in the Oslo Health Study. Furthermore, our study reported higher levels of PM_10_ and PM_2.5_ (53.0 μg/m^3^ and 23.1 μg/m^3^) than those reported in the Oslo Health Study (14.9 μg/m^3^ and 12.5 μg/m^3^). The effects of the different bone health indicators: self-reported forearm fracture and bone mineral density vs. osteoporosis diagnosis and treatment have to consider the associations between air pollution and osteoporosis incidence [10].

According to level of urbanization, bone health tends to be indicated by lifestyle differences in the amount and type of physical activity and dietary intake of calcium and vitamins. However, lifestyle might not influence BMD directly or relatively influence bone size [27]. Additionally, osteoporosis diagnoses have been confirmed earlier in rural areas than in urban areas [28]. We found a more hazardous effect of PM_10_ on osteoporosis incidence in less urbanized areas. The composition of air pollution caused by burning wood and coal in less urbanized areas should be controlled to compare according to urbanization level [9]. Furthermore, the levels of vitamin D in indoor/outdoor life and air pollutants should be considered when these results are interpreted.

This study reported the associations after adjusting for a wide range of comorbidities that contribute to osteoporosis. Peptic ulcer disease is an independent risk factor for osteoporosis regardless of calcium intake [29]. Additionally, chronic diseases including cardiovascular disease [30], diabetes mellitus [31], liver disease [32], musculoskeletal deformity [33], depression [34], and pulmonary disease [35], which had been diagnosed before osteoporosis and had similar mechanisms, were controlled. Therefore, PM_10_ can be understood as an independent risk factor for osteoporosis, although this study did not measure directly patient lifestyle or bodyweight.

This study had several limitations. First, we defined the osteoporosis group based on ICD-10 codes and prescription data and did not include BMD measurement, which is a marker for bone resorption or formation, or serum vitamin D level. Therefore, it is possible that the study subjects do not accurately reflect data for real patients [3, 4]. However, the prevalence of osteoporosis defined by BMD measurement in the last survey was similar to the results of this study [36]. Second, our data lacked information on the severity of osteoporosis and risk factors, including body mass index, diet pattern, estrogen replacement, smoking, alcohol consumption, education, and physical activity. This study was performed based on population groups and did not reflect individual factors. Additionally, the effects of meteorological variables on air pollution were important to consider because negative associations of temperature, wind speed, and humidity with PM_10_ have been confirmed [37]. Although the actual impacts of meteorological variables on air pollution are unclear in current long-term analysis because the relative changes are minimal [38], further studies considering various meteorological factors are needed. Third, individual concentrations of air pollution are not available to estimate the effect in the same administrative districts. Consequently, the air pollution level of an administrative district could be influenced by the adjacent sources of air pollution (e.g., transportation, industry). Therefore, this study has limitations that do not reflect individual exposure concentrations, and there are challenges with applying these data to interpolation analysis. Fourth, we targeted only newly diagnosed osteoporosis cases to assess the effects of air pollution and were not able to account for the associations between osteoporotic fracture, osteosarcopenia, and air pollution. Finally, it is important to consider biases that can arise from exclusion of subjects due to non-matching because of the location of air pollution surveillance stations and residence area. In spite of these limitations, this study contributed to the understanding and clinical considerations for bone health because there are only a limited number of studies on the relationship between air pollution and osteoporosis in the general population with long-term exposure to PM_10_, NO_2_, SO_2_, and CO.

In conclusion, we found an association between long-term exposure to PM_10_ and newly diagnosed osteoporosis in Korean adults aged ≥40 years using nationwide population-based data and air pollution measurements from nationwide surveillance stations. The association between exposure to PM_10_ and incidence of osteoporosis differed according to participant characteristics. However, PM_2.5_, NO_2_, CO, and SO_2_ did not affect the incidence of osteoporosis. The development of environmental policies to control air pollution can improve human health, including bone health. Further studies are warranted to identify the associations of severity of osteoporosis with treatment effect and air pollution, considering individual confounding factors.

## Supplementary Information

**Additional file 1.**

## Data Availability

The datasets used and/or analyzed during the current study are available from the corresponding author on reasonable request and with the National Health Insurance Sharing Service’s permission.
