# Supplementary material for: Incidence of osteoporosis and ambient air pollution in South Korea: a population-based retrospective cohort study
Source: BMC Public Health. 2021 Oct 6;21:1794. doi: 10.1186/s12889-021-11866-7 (PMC8493748; doi:10.1186/s12889-021-11866-7)
Supplement: Supplementary file 1 — Additional file 1. [file 12889_2021_11866_MOESM1_ESM.docx]

**Supplementary figure**. The hazard ratios (HR) and 95% confidence intervals of osteoporosis according to the concentration of PM_10_ in Korea


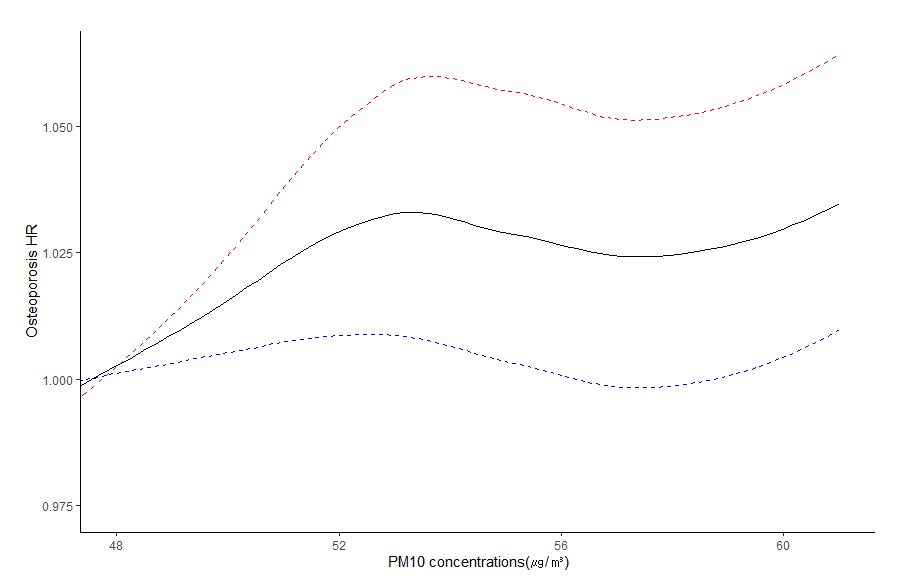


**Supplementary** **table.** The correlation coefficients between air pollutants and meteorological data

|  | PM_2.5_ | PM_10_ | NO_2_ | SO_2_ | CO | Temperature | Rainfall | Wind speed |
| --- | --- | --- | --- | --- | --- | --- | --- | --- |
| PM_2.5_ | 1 | 0.761* | 0.187* | 0.288* | 0.202* | -0.276* | -0.310* | -0.089* |
| PM_10_ |  | 1 | 0.166* | 0.443* | 0.196* | -0.236* | -0.009 | -0.089* |
| NO_2_ |  |  | 1 | 0.512* | 0.714* | -0.402* | -0.210* | -0.379* |
| SO_2_ |  |  |  | 1 | 0.473* | -0.132* | -0.120* | -0.200* |
| CO |  |  |  |  | 1 | -0.441* | -0.102 | -0.518* |
| Temperature |  |  |  |  |  | 1 | 0.035 | 0.810* |
| Rainfall |  |  |  |  |  |  | 1 | -0.007 |
| Wind speed |  |  |  |  |  |  |  | 1 |

*p<0.05

Particulate matter <10 μm (PM_10_), Nitrogen dioxide (NO_2_), Sulfur dioxide (SO_2_), and Carbon monoxide (CO) were measured between 2002 − 2015. Particulate matter < 2.5 μm (PM_2.5_) was measured in 2015.

Temperature, rainfall, and wind speed were shown between 2003 – 2015 from the Korea Meteorological Administration, Seoul, Korea. https://data.kma.go.kr/climate/extremum/selectExtremumList.do?pgmNo=103
